# *Caenorhabditis elegans* in microgravity: An omics perspective

**DOI:** 10.1016/j.isci.2023.107189

**Published:** 2023-06-20

**Authors:** Amanda Scott, Craig R.G. Willis, Masafumi Muratani, Atsushi Higashitani, Timothy Etheridge, Nathaniel J. Szewczyk, Colleen S. Deane

**Affiliations:** 1 Department of Biomedical Sciences, Heritage College of Osteopathic Medicine, Ohio University, Athens, OH, USA; 2 School of Chemistry and Biosciences, Faculty of Life Sciences, University of Bradford, Bradford, UK; 3 Transborder Medical Research Center and Department of Genome Biology, Faculty of Medicine, University of Tsukuba, Tsukuba, Ibaraki 305-8575, Japan; 4 Graduate School of Life Sciences, Tohoku University, Sendai, Japan; 5 Department of Sport and Health Sciences, College of Life and Environmental Sciences, University of Exeter, Exeter, UK; 6 Ohio Musculoskeletal and Neurological Institute, Ohio University, Athens, OH, USA; 7 Human Development & Health, Faculty of Medicine, University of Southampton, Southampton General Hospital, Southampton, UK

**Keywords:** Omics, Model organism, Space sciences

## Abstract

The application of omics to study *Caenorhabditis elegans* (*C. elegans*) in the context of spaceflight is increasing, illuminating the wide-ranging biological impacts of spaceflight on physiology. In this review, we highlight the application of omics, including transcriptomics, genomics, proteomics, multi-omics, and integrated omics in the study of spaceflown *C. elegans*, and discuss the impact, use, and future direction of this branch of research. We highlight the variety of molecular alterations that occur in response to spaceflight, most notably changes in metabolic and neuromuscular gene regulation. These transcriptional features are reproducible and evident across many spaceflown species (e.g., mice and astronauts), supporting the use of *C. elegans* as a model organism to study spaceflight physiology with translational capital. Integrating tissue-specific, spatial, and multi-omics approaches, which quantitatively link molecular responses to phenotypic adaptations, will facilitate the identification of candidate regulatory molecules for therapeutic intervention and thus represents the next frontiers in *C. elegans* space omics research.

## Graphical abstract

**Figure:**
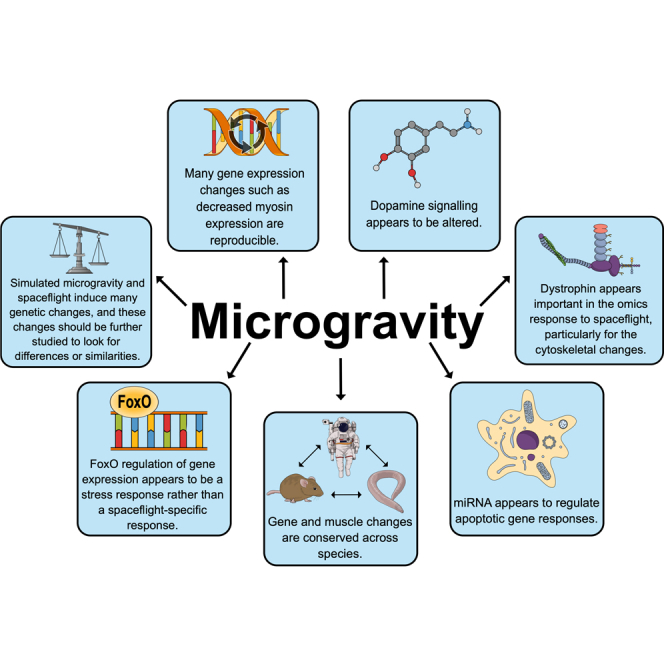

## Introduction

Since Yuri Gagarin first traveled to space in 1961, human spaceflight activity has grown rapidly. One of the most notable events to date was the 1998 launch of the International Space Station (ISS), a lower Earth orbiting research platform, which allows astronauts to live and work in space for up to a year at a time.^1^ The microgravity environment of space presents an extreme environmental stressor, which induces a multitude of physiological maladaptations including, but not limited to, skeletal muscle and bone loss, cardiovascular decline, immune compromise,^2^ neuronal damage, and decreased gray matter volume.^3^ These resemble some of the adverse health changes associated with chronological aging on Earth, and additionally serve as an accelerated model of other non-communicable health concerns such as diabetes, cardiovascular disease, and obesity.^4^ As people look to travel further into space and for longer periods than ever before—owing to the rise in commercial spaceflight and increasingly lower cost access to space—understanding the mechanistic underpinnings of spaceflight-induced maladaptation is essential to developing effective countermeasures that promote safe space travel and also benefit health on Earth.

Human and rodent studies have provided invaluable insight into the mechanistic regulation of the microgravity phenotype; however, they are limited by significant operational (e.g., crew time, human/rodent numbers, and sample feasibility) and economical (e.g., high costs) constraints.^5^^,^^6^ Alternative model organisms are, therefore, required to achieve the experimental power necessary to draw conclusions on the causes of space-induced health decline. *Caenorhabditis elegans* (*C. elegans*) is a non-parasitic, microscopic (∼0.25–1 mm) nematode characterized by a short lifespan (∼3 weeks), large brood size, fully sequenced genome, multiple evolutionary conserved mechanisms, ease of culture, and low expense,^7^ presenting a primary biologically relevant model for spaceflight research that is relatively cheap and feasible. In fact, *C. elegans* spaceflight experiments have been conducted since the time of the space shuttle. Previous studies have shown that *C. elegans* can mate, reproduce, and undergo embryonic development during spaceflight, and they are also susceptible to radiation-induced mutations during spaceflight.^8^^,^^9^ However, since these experiments were performed on ordinary agar medium, the effects of strong hydrostatic pressure adhesion between the *C. elegans* and the agar surface could not be ruled out. Therefore, in examining the effects of microgravity, a liquid culture was devised. The first space experiment to test the difference between liquid and agar media could not be analyzed due to an unforeseen accident on Columbia’s return. However, the *C. elegans* were found alive a few days later, protected by a sturdy container.^10^ Since then, our space experiments have been conducted using CeMM (*C. elegans* Maintenance Medium)^11^ and *E. coli* OP-50 liquid media. In addition, *C. elegans* permit the use of genetic and molecular tools (e.g., genetic mutants), to dissect specific biological pathways and trial emerging countermeasures, such as the efficacy of preclinical drugs.^12^ Since *C. elegans* display high molecular, metabolic, and physiologic orthology with people, with as much as 83% of the *C. elegans* proteome sharing homologous genes with humans,^13^ they serve as efficient model organisms to understand (human) disease processes. For example, their use as a biomedical research model organism has been invaluable in the study of Alzheimer disease and Duchenne muscular dystrophy.^14^ Specific to spaceflight, *C. elegans* successfully live in microgravity^15^ and display reduced movement capacity,^16^ analogous to the decline in muscle function observed in astronauts post-flight,^17^ demonstrating the suitability of *C. elegans* as a model organism to study the effects of spaceflight on physiology. Therefore, using *C. elegans* to uncover the precise molecular mechanisms regulating adaptations to microgravity can significantly accelerate our understanding of the mechanisms of spaceflight-induced physiological decline in humans.

Recently, there has been an exponential increase in the use of “omics” technologies and informatic tools to probe the etiology of, and develop countermeasures for, pathological states,^18^ including spaceflight.^19^ The application of omics seeks to establish the sum of the parts within a biological system, rather than identifying individual components, promoting a holistic rather than reductive path of biological study.^20^ In addition to the beneficial “systems biology” approach, omics can capitalize on pre-existing biological samples (i.e., samples that were collected before the omics “boom” and “bio-banked” samples), which are particularly rare in the context of spaceflight. Recently, the National Aeronautics and Space Administration (NASA) launched project “GeneLab” to allow free and unrestricted access to omics data from spaceflight experiments to researchers all over the globe.^21^ The success of this open-science model has maximized the use of scarce spaceflight biological samples and led to several discoveries otherwise not possible, such as the identification of spaceflight CO_2_ levels as a confounding variable in the animal enclosure modules used in rodent experiments.^22^ More recently, the European Space Agency (ESA)-funded Space Omics Topical Team has supported new and ongoing ESA scientific community activities that focus on the application and exploitation of omics research in space.^23^^,^^24^ Indeed, omics technology has been applied to spaceflight research using *C. elegans*, leading to a more complete understanding of biological processes that occur in response to spaceflight, although these works remain to be consolidated.

The aim of this narrative review is, therefore, to summarize the literature pertaining to the use of *C. elegans* as a model organism for spaceflight-related research with a specific focus on publications that have applied omics technologies to uncover the mechanistic basis of the microgravity phenotype, thus highlighting how the extensive use of *C. elegans* in spaceflight research has constructed a more coherent picture of the biological response to spaceflight. To do this, we provide an overview of the available omics literature, including hyper-, micro-, and simulated gravity scenarios, highlighting key biological themes and, where relevant, highlight how the molecular flight-responsive signatures in worms correspond with known molecular and/or functional adaptations reported in higher animals and humans. We also summarize the impact of the *C. elegans* space omics literature to date and highlight what we believe are the next frontiers in *C. elegans* space omics research. Although previous reviews have appreciated the use of *C. elegans* as a model organism for spaceflight research, these reviews have either not focused on omics^25^ or are non-comprehensive due to geographical constraints.^24^ Thus, global efforts combining *C. elegans* and omics in space remain to be detailed.

## Omics investigations of *C. elegans* in microgravity

**Figure 1:**
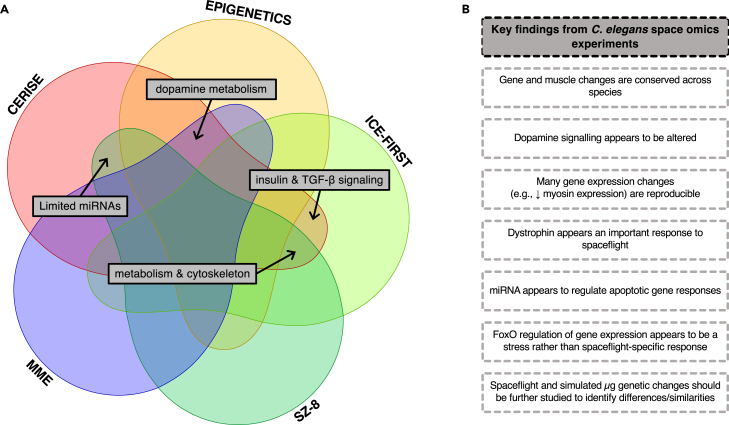
Key insights from *C. elegans* space omics experiments (A) Venn diagram depicting reproducible gene expression signatures from multiple microgravity experiments in *C. elegans*. (B) A summary of the key findings from *C. elegans* space omics experiments.

To date, the majority of omics spaceflight research in *C. elegans* is transcriptomic in nature, with far fewer proteomic investigations and comparative omics analysis (summarized in Tables 1, 2, and 3, respectively). Together, these investigations have revealed robust metabolic and neuromuscular biological themes, discussed herein (Figure 1).

**Table 1:** Transcriptomic investigations of *C. elegans* in the context of microgravity

Authors	Spaceflight Mission/s	Omics Analysis Platform	Key Outcomes	Take Home Message
Celen et al.^26^	CERISE Simulated microgravity	RNA-seq	- Simulated microgravity affects the sphingolipid signaling pathway and the longevity-regulating insulin/IGF-1 pathway - 118 genes were identified as commonly differentially expressed in *C. elegans* exposed to either simulated microgravity or spaceflight	Detrimental effects from spaceflight are replicable in simulated microgravity and pose long-term health concerns
Sudevan et al.^27^	CERISE EPIGENETICS MME Simulated microgravity	Microarray	- Spaceflown *C. elegans* consistently display decreased *comt-4* gene expression (a catechol-*O*-methyltransferase dopamine degradation enzyme)	Targeting the dopamine system may be a viable and realistic therapeutic to promote safe space travel
Sun et al.^28^	Simulated microgravity	RNA-seq	- Multiple intestinal long non-coding RNAs were increased/decreased by simulated microgravity - Knockdown of a candidate long non-coding RNA and simulated microgravity led to the dysregulation of 43 genes	Long non-coding RNAs are involved in regulating the response to simulated microgravity
Higashitani et al.^29^	EPIGENETICS	Microarray	- 39 genes expressed in *C. elegans* in response to microgravity are suppressed by the action of HDA-4	(At least some) transcriptional changes caused by microgravity are epigenetically controlled
Sun et al.^30^	Simulated microgravity	miRNA-Seq	- 19 miRNAs are dysregulated by exposure to simulated microgravity, many of which served to protect against the toxicity of microgravity	miRNA regulation can be protective against the negative effects of simulated microgravity
Tee et al.^31^	Simulated microgravity	Microarray	- 1 gene was downregulated in nematode growth medium-cultured *C. elegans* exposed to simulated microgravity - 6 genes were upregulated and 2 downregulated in *C. elegans* exposed to Maintenance Medium during microgravity - 5 of the upregulated genes encoded non-coding RNAs	Simulated microgravity on a single generation of *C. elegans* did not induce major transcriptional changes
Gao et al.^32^	SZ-8	Microarray microRNA microarray	- Protein phosphorylation/dephosphorylation is receptive to microgravity and space radiation - Microgravity impacts transcription depending on the dystrophin gene (*dys-1*) - Loss of function of *ced-1* (apoptotic gene) leads protective responses to space radiation	Microgravity elicits transcriptional changes in *C. elegans* during short-duration spaceflight
Higashibata et al.^33^	CERISE	Microarray	- Downregulation of muscle-related genes (such as the myosin heavy chains, troponins, and intermediate filaments) and metabolic genes in spaceflown samples	Gene changes reproducible across spaceflights
Zhao et al.^34^	SZ-8	Microarray	- 86 differentially expressed genes were identified in response to space, and were mainly related to oxidative phosphorylation	Microgravity was the main factor to induce the biological effects in the level of gene expressions, rather than space radiation
Gao et al.^35^	SZ-8	Microarray microRNA microarray	- Spaceflight increased the number of differentially expressed miRNAs - Space conditions increased changes in the expression of miRNAs that may impact development, energy metabolism, apoptosis, and signaling transduction	miRNAs are likely involved in apoptosis during short-term spaceflight
Gao et al.^36^	SZ-8	Microarray microRNA microarray	- Double the number of transcripts were significantly altered in the spaceflight environment compared with space radiation alone - The majority of alterations were related to protein amino acid dephosphorylation and histidine metabolic and catabolic processes - cel-miR-81, cel- miR-82, cel-miR-124, and cel-miR-795 were predicted to regulate DNA damage response	Microgravity, in the presence of radiation, enhances the DNA damage response in *C. elegans*
Xu et al.^37^	SZ-8	Microarray microRNA microarray	- 23 miRNAs were altered in spaceflight - Most putative target genes of the altered miRNAs were predicted to be involved in developmental processes - cel-miR-52, -55, and −56 of the miR-51 family were sensitive to space environmental stressors	*C. elegans* respond to spaceflight by altering miRNAs expression
Etheridge et al.^38^	CERISE	Microarray microRNA	- The expression of the vast majority of microRNAs is not affected by microgravity - RNAi effectively silenced transgenic and endogenous genes in the gonad - RNAi against lysosomal protease genes prevented muscle protein degradation	RNAi works as effectively in the spaceflight environment as on Earth within multiple tissues
Selch et al.^39^	ICE-FIRST	Microarray	- Normal developmental timing, apoptosis, and DNA repair transcriptional signature - Altered muscle developmenttranscriptional signature - Spaceflown *C. elegans* undergo a metabolic shift, which may be underlaid by insulin and/or TGF-β signaling	*C. elegans* can be used to study the effects of altered gravity
Higashibata et al.^40^	ICE-FIRST	Microarray	- Microgravity upregulated genes related to early embryogenesis - Microgravity downregulated genes related to locomotion	Microgravity regulates genes related to locomotion, early embryogenesis, and regeneration in *C. elegans.*
Higashibata et al.^16^	ICE-FIRST	Microarray	- Myosin heavy chain isoforms in the body wall muscles (isoforms A & B) and the pharyngeal muscles (isoforms C & D) decreased in expression in *C. elegans* developed in space, relative to ground controls - Expression of transcription factors controlling the expression of the myosin heavy chain isoforms was also decreased - Genes encoding troponins and tropomyosins were also decreased	*C. elegans* muscle development is altered in spaceflight, which may contribute to spaceflight-induced muscle atrophy

CERISE, *C. elegans* RNAi Space Experiment; EDL, extensor digitorum longus; EPIENETICS, The effect of microgravity on the epigenetic modification in *C. elegans*; ICE-FIRST, FIRST International *C. elegans* Experiment in space; IGF-1, Insulin-like growth factor 1; ISS, International Space Station; MME, Molecular Muscle Experiment; SZ-8, Shenzhou-8; TGF-β, transforming growth factor-beta.

**Table 2:** Proteomic investigations of *C. elegans* in the context of microgravity

Authors	Spaceflight	Omics Analysis Platform	Key Outcomes	Take Home Message
Higashibata et al.^33^	CERISE	iTRAQ	- 16 upregulated proteins, with many related to protein synthesis - 43 downregulated proteins, many being cytoskeletal and metabolic proteins	Protein changes reproducible across spaceflights
Higashibata et al.^41^	ICE-FIRST	2D gel electrophoresis and MALDI-TOF MS	- ∼100 proteins identified - Spaceflight reduced paramyosin and aconitase expression - Spaceflight reduced the expression of troponin T, but increased the phosphorylation of troponin T	Spaceflight influenced the expression of muscle-related proteins
Higashibata et al.^40^	ICE-FIRST	2D gel electrophoresis	- >1000 total protein spots and ∼200 phosphoprotein spots detected - 10%–15% significantly increased or decreased in spaceflown samples - Several phosphoprotein spots also significantly reduced in spaceflown samples	Spaceflight impacts proteome

CERISE, *C. elegans* RNAi Space Experiment; ICE-FIRST, FIRST International *C. elegans* Experiment in space; ISS, International Space Station; iTRAQ, isobaric tags for relative and absolute quantitation; MALDI-TOF, matrix-assisted laser desorption/ionization-time of flight; MME, Molecular Muscle Experiment; MS, mass spectrometry; 2D, 2 dimensional.

**Table 3:** Comparative omic investigations of *C. elegans* in the context of microgravity

Authors	Spaceflight Mission/s	Omics Analysis Platform	Key Outcomes	Take Home Message
Cahill et al.^12^	ICE-FIRST	Microarray	- *C. elegans* transcriptional response to spaceflight demonstrates greater similarity with mouse EDL (fast-twitch muscle) compared to the soleus (slow-twitch muscle) - There was a similar increase in proliferation-related genes, cAMP signaling	*C. elegans* results might be able to be extrapolated to mammalian results
Willis et al.^5^	Microgravity: ICE-FIRST CERISE Hypergravity	Microarray	- Most common gene signatures between hypergravity and microgravity were similarly upregulated (ubiquitin-mediated catabolism, actin cytoskeleton regulation, cell cycle processes) or downregulated (immune system regulation) - “Metabolic regulation” displayed opposing regulation in response to hypergravity versus microgravity	Hypergravity and microgravity elicit neuronal and metabolic adaptations
Leandro et al.^42^	ICE-FIRST	Microarray	- Only 6 genes were commonly downregulated in *C. elegans* and *Drosophila melanogaster* - Although, the number of genes that could be compared was reduced to only ∼20% of the comparative genome, due to ortholog-related challenges - The 6 genes were associated with metabolic and neuromuscular signaling	Very few genes are commonly regulated between *C. elegans* and *Drosophila melanogaster* by spaceflight

cAMP, cyclic adenosine monophosphate; CERISE, *C. elegans* RNAi Space Experiment; EDL, extensor digitorum longus; ICE-FIRST, FIRST International *C. elegans* Experiment in space.

### Metabolic alterations in microgravity

Both transcriptomic and proteomic investigations have been applied in an effort to understand the biological response of *C. elegans* to spaceflight, which have revealed robust metabolic alterations. In fact, three independent spaceflight missions, the FIRST International *C. elegans* Experiment in space (ICE-FIRST), *C. elegans* RNA Interference in Space Experiment (CERISE), and Shenzhou-8 (SZ-8), have all found altered metabolism in flight (Figure 1). The first investigation into the transcriptional responses in spaceflown worms was ICE-FIRST in 2006. Using microarray analysis, the authors demonstrated a metabolic shift occurring mainly in the intestines that could be underlaid by altered TGF-β and insulin signaling (Figure 1).^39^ Considering the possible implications for altered endocrine signaling due to altered metabolism of lipids as well as alterations to innate immunity, these changes to TGF-β and insulin signaling warrant further study.^32^^,^^39^

Just as microgravity is associated with consistent insulin signaling changes, simulated microgravity also elicits reproducible insulin signaling changes. For example, a recent study looking at simulated microgravity-exposed *C. elegans* found altered expression in the insulin/IGF-1 signaling pathway.^26^ Another study found that simulated microgravity-exposed *C. elegans* demonstrated decreased expression of *linc-50*, an intestinal long non-coding RNA which appears to target FoxO transcription factor DAF-16, which is part of the insulin signaling pathway downstream of the insulin receptor/DAF-2.^28^

Another element of the ICE-FIRST effort was also the first known proteomic investigation into spaceflown *C. elegans*, which found that spaceflight reduced aconitase expression; a citric acid cycle enzyme responsible for citrate-isocitrate conversion, which could affect ATP generation (Figure 1).^41^ Other studies have demonstrated a similar pattern of alterations to genes and processes involved in the generation of ATP. For example, one such study found 86 differentially expressed genes in response to spaceflight, which were related to the process of mitochondrial respiration, which is the final step of the process of cellular respiration that generates ATP.^34^

Subsequent proteomic and transcriptomic analysis of spaceflown *C. elegans* from the 2009 CERISE experiment also displayed alterations in metabolism on two fronts. First, the mitochondrial anchorage protein, ANC-1, was found to have decreased levels of expression.^33^ ANC-1 is involved in nuclear anchorage and positioning, as well as the positioning of mitochondria and the endoplasmic reticulum.^43^ Mutations to ANC-1 negatively affect the health of *C. elegans*, with ANC-1 mutants displaying smaller body sizes, changes to the shape of the nucleus, fragmentation of the endoplasmic reticulum, and movement of the nucleus and endoplasmic reticulum during body movement.^43^ Second, multiple metabolic proteins in all stages of cellular respiration, including glycolysis and the glyoxylate cycle, gluconeogenesis, and the electron transport chain, were found in significantly reduced levels (Figure 1).^33^

The launch of the NASA GeneLab database^21^ and more recent initiatives such as the ESA-funded Space Omics Topical Team^23^^,^^24^ has allowed access to vast amounts of spaceflight-related omics data, including that from *C. elegans*. Such access facilitates comparative omics investigations, that is using multiple datasets from different conditions/species to identify reproducible omics profiles, maximizing the scientific return on investment. In one such comparative analysis, the authors aimed to understand whether the transcriptional profiles of microgravity are the inverse of the hypergravity expression pattern, a hypothesis worthy of investigation considering the divergent physiological adaptations to microgravity versus hypergravity.^5^ The authors found that while most common gene signatures were similarly up- or downregulated, genes pertaining to the Gene Ontology term “metabolic regulation” displayed opposing regulation, being upregulated in hypergravity and downregulated in microgravity.^5^ Downstream network analysis identified a tyrosine degrading enzyme, *hpd-1*, as the most highly connected hub gene providing a candidate for future therapeutic targeting.^5^

Research into similarities in the response to spaceflight between organisms has demonstrated that the subsequent metabolic changes are not unique to *C. elegans.* One study used comparative omics to explore spaceflight gene expression changes between *Drosophila melanogaster* and *C. elegans* in order to understand how similarly or dissimilarly these organisms respond to spaceflight.^42^ Surprisingly, only 6 genes were commonly downregulated in both organisms, several of which were associated with metabolic signaling, although due to ortholog-related challenges the number of genes that could be compared was reduced to roughly only 20% of the comparative genome.^42^

Examined together, all of the studies discussed previously demonstrate a clear pattern of metabolic changes in response to spaceflight and microgravity, affecting primarily the mitochondria and the processes involved in the generation of ATP. Decline in mitochondrial ATP production occurs naturally with age, and mitochondrial dysfunction has been found in humans with a variety of health conditions, including type 2 diabetes mellitus and insulin resistance.^44^ Further, mitochondrial dysfunction has been linked as being both a cause and consequence of muscle atrophy due to muscle disuse.^45^^,^^46^ These alterations in mitochondria could also partly explain phenotypes in other high energy-consuming tissues such as neurons.^47^ Therefore, understanding these metabolic changes induced in spaceflight contributes to understanding the cause of muscle decline and alterations in other tissue systems observed in astronauts.

### Muscular and neuromuscular alterations in microgravity

Omics analysis of microgravity samples has also highlighted robust muscular and neuromuscular changes in spaceflown *C. elegans*. For example, spaceflown *C. elegans* from ICE-FIRST were characterized by altered muscle development, which was accompanied by corresponding declines in myosin heavy chain isoform gene expression^16^ and locomotion-related genes (Figure 1).^40^ It should be noted that the ICE-FIRST experiments were carried out in a chemically defined medium, which permitted the study of the effects of surface tension in spaceflight, but significantly alters *C. elegans* development akin to dietary restriction.^33^^,^^48^ Proteomic analysis from the same spaceflight mission (ICE-FIRST) found the core thick filament protein, paramyosin, was reduced in spaceflown samples.^41^ Additionally, spaceflight reduced troponin T, which binds tropomyosin and tethers the troponin complex to the thin filament, but increased troponin T phosphorylation.^41^ These data suggest that these genes and proteins may play a key role in the maintenance of muscle structure and function, and that their expressional change may be involved in spaceflight-induced muscle decline.

Utilizing quantitative isobaric tag for relative and absolute quantitation methodology, the follow-on CERISE experiment found that spaceflown *C. elegans* displayed an increase in 16 proteins mostly related to protein synthesis, including ribosomal proteins and translation elongation factors, and a decrease in 43 proteins, predominantly cytoskeletal, muscular, and mitochondrial related.^33^ Spaceflight-induced declines in muscular (e.g., UNC-15 (paromysin)) and cytoskeletal (e.g., ACT-5 (actin)) gene and protein expression have been shown, suggesting that the observed downregulation in these genes translates to reduced expression of the encoded protein.^33^ Importantly, physiological declines, such as reduced body length and altered movement, were also present suggesting these translational expression changes are physiologically meaningful. Of particular importance, the downregulation of myosin heavy chain and paramyosin genes reproduced earlier proteomic observations,^41^ providing confirmation that the observed proteomic changes were indeed due to microgravity.

Two recent studies highlighted potential therapeutic avenues to target in order to offset muscle decline in space. Sudevan et al. demonstrated that spaceflown *C. elegans* from 3 independent spaceflight missions consistently displayed decreased *comt-4* gene expression (Figure 1), which is a catechol-*O*-methyltransferase dopamine degradation enzyme.^27^ Thus, targeting the dopamine system may be a viable and realistic therapeutic to promote health in space. Another study found that simulated microgravity-exposed *C. elegans* which overexpressed one of three miRNAs (*mir-54*, *mir-354*, or *mir-2208*) experienced a lower level of effect on locomotion behavior, suggesting miRNA expression may have a role in resisting the detrimental effects of microgravity.^30^

Additionally, *C. elegans* shows potential as a model organism to explore the neuromuscular changes found in other animals exposed to spaceflight and microgravity. In one comparative analysis study, it was demonstrated that *C. elegans* transcriptional response to spaceflight demonstrates greater similarity with mouse extensor digitorum longus (fast-twitch muscle) compared to the soleus (slow-twitch muscle).^12^ Consistent with these observations, a shift from slow-twitch fiber toward fast-twitch fiber was seen in the soleus muscle in spaceflown mice.^49^ This may suggest that *C. elegans* results can be extrapolated to mammalian results; however, the caveat of this analysis is that whole *C. elegans* (i.e., muscle, neurons, gut, and reproductive tissue) were utilized and so the transcriptional signature is not isolated to *C. elegans* muscle.^12^

There are also spaceflight experiments not yet published that are exploring neuromuscular changes during spaceflight and generating transcriptomic datasets. For example, in 2018, there was the launch of the first UK-led experiment to the ISS, the Molecular Muscle Experiment (MME), which aims to demonstrate the precise mechanisms underlying muscle decline in space and to explore the efficacy of novel therapeutic interventions aimed at insulin signaling and muscle attachment complexes for offsetting spaceflight-induced muscle decline.^50^ More recently in 2021, there was the follow-up Molecular Muscle Experiment 2 (MME2), which aims to confirm past spaceflight-induced gene expression changes (i.e., MME) and trial the efficacy of pharmacological interventions aimed at improving mitochondrial health and neuromuscular signaling. Through collaboration with NASA GeneLab, samples from MME and MME2 have been subjected to RNA-sequencing and are undergoing analysis.

Together, the research discussed previously has highlighted many muscular and neuromuscular alterations in response to spaceflight and microgravity, providing the mechanistic basis for future studies of how microgravity elicits muscle adaptations in *C. elegans*.^33^ Importantly, some of these reproducible gene expression changes observed in spaceflown *C. elegans*, such as decreased myosin expression, are also observed in rodents and humans.^51^ Thus, spaceflight-induced changes in *C. elegans* gene expression are conserved in higher organisms and may explain the muscle decline observed in astronauts.

### MicroRNA and epigenetic alterations in microgravity

Transcriptional understanding of spaceflight (patho)physiology has been built upon by researchers based in China, following the SZ-8 mission. These authors focused on understanding the role of microRNAs (non-coding RNAs that control gene expression via the destabilization of messenger RNA targets at the post-transcriptional level and via translational inhibition) in response to microgravity and space radiation versus space radiation in isolation.^37^ The authors found microRNAs that are altered in spaceflight,^37^ more so than in response to radiation alone,^35^ are involved in developmental, DNA damage, apoptotic, and metabolic responses.^34^^,^^35^^,^^36^^,^^37^ The authors found that microRNAs are involved in apoptotic gene expression due to the stressors of spaceflight (Figure 1).^36^ Additionally, another study found that *C. elegans* exposed to microgravity demonstrated greater non-coding RNA gene expression.^31^ The data from these studies suggest that epigenetic changes occur with microgravity exposure.^31^

Microgravity has also been shown to alter DNA repair genes and the regulatory microRNAs. In a recent integrative analysis combining transcriptional and microRNA datasets from 3 microgravity experiments that each included spaceflight, spaceflight control with a 1-G centrifugal device, and ground control conditions, Zhao and colleagues reported significant alterations in the expression of DNA repair genes in response to microgravity.^52^ Their integrative analysis approach allowed the identification of microRNA’s as potential post-transcriptional regulators that regulate the DNA repair gene expression in microgravity conditions. For example, under spaceflight/ground control conditions, the authors reported that 6 differentially expressed miRNAs (cel-miR-90, cel-miR-124, cel-miR-124, cel-miR-355, cel-miR-795, and cel-lsy-6) potentially regulate the expressions of 4 DNA repair genes (*rfs-1*, *rcq-5*, *xpc-1*, *and mnat-1*),^52^ demonstrating the power of omic analysis to provide insight into the altered DNA response in microgravity conditions.

Considering robust transcriptional changes occur in spaceflown *C. elegans*, it has been suggested that epigenetic changes may also occur since epigenetic modifications, such as DNA methylation and histone modifications, regulate gene expression.^29^ Despite this sound hypothesis, research into the epigenetic responses of spaceflown *C. elegans* has only recently begun. In the only relevant study known to date, microarray analysis revealed that 39 genes expressed in *C. elegans* in response to microgravity are suppressed by the action of HDA-4.^29^ This suggests that microgravity-induced transcriptional changes are potentially, in part, epigenetically controlled, thus calling for further research into the epigenetic programs underpinning microgravity adaptations, especially considering the potential heritability of any epigenetic changes.^53^ The potential intergenerational effects of spaceflight have already been reported in mice, where one study found that the offspring of space-traveled mice demonstrated altered expression of genes in the liver.^54^ Further study will build a deeper understanding of the effects of microgravity on the epigenome and the potential ramifications for any potential future offspring of spaceflown organisms and astronauts.

## Insights and impact of *C. elegans* space omics work

As elucidated previously, space omics studies in *C. elegans* have generated several valuable insights on the molecular regulation of spaceflight maladaptation which, in turn, stand to beneficially impact future translatable research efforts toward better understanding and counteracting spaceflight-induced health declines. For instance, transcriptomic analyses highlight strong overlap of dysregulated genes in *C. elegans* from different spaceflight missions, despite variant experimental factors.^5^^,^^33^ These reproducible findings highlight the utility of *C. elegans* as a prime model organism for *robust* spaceflight omics investigation and in particular future efforts aimed at ameliorating impaired molecular responses to spaceflight (the MME and MME2 missions as two examples). Particularly evident from previous worm space omics research are reproducible expression changes in neuronal/muscular and metabolic genes.^5^^,^^33^ Given that worms in space might feasibly tract neuromuscular changes in gene expression of higher organisms in space,^12^ omics work to date thus also pinpoints *C. elegans* as an especially valuable model for understanding molecular causes of and developing countermeasures to declines in astronaut muscle health.

Interestingly, dysregulation of key muscle genes is evident even when spaceflown worms are compared to onboard 1-G control worms,^33^ implicating that such expression changes are the consequence of microgravity more so than increased radiation exposure. That mechanical load is the partial driver of muscle changes in space is further supported by other previous worm space microarray analyses, wherein dystrophin (a highly mechanosensitive molecule that plays a key role in maintaining muscle structure/function)^55^ was shown to be important in the omics response to spaceflight, particularly with respect to neuromuscular/cytoskeletal changes.^56^ In fact, transcriptional changes in worms generally appear more widespread in the spaceflight environment than with space radiations alone,^35^ with *C. elegans* work thus indicating that microgravity is the more predominant effector of spaceflight omics changes. More specifically, the space radiation dose on the ISS is ∼0.5–1 mSv per day, including ∼50 charged particles/sr/cm2 with high linear energy transfer (>10 keV/μm).^57^^,^^58^^,^^59^ Since an adult nematode *C. elegans* is ∼1 mm long and 0.05 mm wide, the probability of a direct hit by charged particles is relatively low. In addition, DNA damage by heavy particle radiation, not only ionizing radiation, is efficiently repaired in meiotic oocytes, which make up the majority of nematode adult gonads, because of their high homologous recombination activity.^60^^,^^61^ Thus, it is challenging to precisely evaluate the effects of space radiation in omics analyses using a large number of *C. elegans* cultured for a short period. Instead, the effects of space radiation on *C. elegans* are mainly studied by examining the frequency of mutations that can be observed in each individual nematode.^8^^,^^62^^,^^63^ Thus, while it appears from studies in *C. elegans* that radiation exposure in lower Earth orbit is not a major contributor to observed omics changes, the effects in higher mammals and humans, as well as the potential effects beyond in lower Earth orbit and the protective effects of the Van Allen belts, render the consequences of spaceflight radiation on omics/biology uncertain.

Omics work in spaceflown worms has also provided several important insights into upstream regulatory mechanisms of spaceflight molecular responses. For example, microarray analysis of spaceflown *C. elegans* implicates a prominence toward insulin signaling regulation of spaceflight-induced expression changes^39^ that is likely to be partly driven by DAF-16/FoxO^25^—though more recent comparative transcriptomic analyses of *C. elegans* suggest that FoxO regulation of gene expression may be a stress response to altered gravity *per se*.^5^ Regardless, given that FoxO signaling is a highly conserved regulatory pathway across species,^64^ such worm omics data should provide a strong basis to accelerate mechanistic understanding of poor spaceflight adaptations of higher organisms.^5^ Indeed, recent evidence supports insulin signaling alterations during spaceflight in higher organisms, including both rodents and astronauts.^65^ Finally, more recent transcriptomic-driven analysis of spaceflown worms suggests that dopamine signaling is also perturbed with microgravity.^27^ Key implications and insights from *C. elegans* space omics work have been further summarized in Figure 1.

## Next frontiers in *C. elegans* space omics

### Linking omics to phenotype

While understanding the molecular responses to microgravity in isolation are important, these will be more informative if they quantitatively link to functional outcomes. Using informatic approaches, authors can, and should, directly relate the *C. elegans* omics signatures to functional outcomes such as movement rate and/or strength, to identify biologically important pathways and molecules regulating the phenotypic responses to microgravity. Ideally, all of these analyses would be performed in the same animals to make firm mechanistic links.^33^ Indeed, such “omics-phenotype” links have been made previously, albeit not in the context of spaceflown *C. elegans*.^66^^,^^67^ This approach can also be taken toward the creation and application of personalized medicine in the treatment of human disease. The potential use of *C. elegans* as a model organism for personalized medicine is already being explored in various ways. One study explored using *C. elegans* as a model for examining variations in metabolism between individuals via the application of metabolomics and comparative genomics, demonstrating differences in the levels of several metabolites between four strains of *C. elegans*.^68^ This demonstrates the use of *C. elegans* as a building block toward understanding the differences in metabolism between individuals within a species.^68^

### Integrated multi-omics

Without doubt, transcriptomics and proteomics are the most common omics analyses performed thus far in *C. elegans* subjected to microgravity conditions. However, microgravity likely affects many other biological layers, therefore the use of other omics can contribute to a more complete understanding of the effects of spaceflight. For example, *C. elegans*-specific metabolomics and lipidomics methodologies are emerging,^69^ providing additional and powerful omics capabilities to probe the pathophysiological effects of spaceflight and reveal the potential of therapeutic interventions. These multiple omics approaches can be integrated (i.e., at the computational analysis stage) to provide a comprehensive understanding of the condition-specific crosstalk between different biological levels, therein providing a better understanding of microgravity-inducing mechanisms and the identification of novel candidates for therapeutic intervention. Indeed, this approach has been taken in *C. elegans* to investigate responses to aging,^70^ starvation,^71^ and antibiotics,^72^ albeit not in the context of spaceflight.

### Tissue-specific and spatial omics

It is notable that the majority of omics work to date has been generated using RNA/protein extracted from whole worms containing all tissue types (i.e., muscles, gut, and neurons) as opposed to individual tissues, and so the tissue-specific transcriptome/proteome responses to altered gravity are yet to be distinguished.^5^ Indeed, it is possible to isolate and sequence individual tissues derived from *C. elegans*,^73^ and since spaceflight can induced a wide variety of maladaptations across many organs, understanding tissue-specific responses to microgravity is necessary in order to precisely underpin regulatory mechanisms and develop targeted and effective countermeasures against spaceflight-induced maladaptations.

Further, conventional microarray and RNA-sequencing approaches do not provide spatial information, and tissue-specific transcriptomics only partially solves this problem since it still takes into account ubiquitously expressed genes (i.e., housekeeper genes).^74^ Spatial transcriptomics is an emerging method to overcome these limitations, which combines RNA-sequencing with serial tissue sectioning^75^ to identify tissue-specific spatiotemporal gene responses. By adapting the RNA tomography approach originally developed for zebrafish,^75^ Ebbing and co-authors were able to successfully apply spatial transcriptomics to *C. elegans*, which was considered a major challenge due to the limited amount of mRNA that can be extracted from individual sections of small organisms.^74^ The authors individually froze and cryosectioned (20 μm) *C. elegans*, followed by RNA extraction and processing using CEL-seq (to overcome the challenges of low input).^76^ Using this combination of tools, the authors were able to detect an average of 16,394 genes in young wild-type adult hermaphrodites, equating to 93% of genes detected in bulk RNA-sequencing experiments,^77^ and they also demonstrated regional expression was highly indicative of function.^74^ Thus, spatial transcriptomics offers a highly sensitive method to generate complex data, and develop deeper understanding of tissue organization, or disorganization, in disease. Spatial transcriptomics has been used to create tissue atlases that have served not only particular use in creating detailed maps of the nervous system of mice but also furthered understanding of important topics in biology like heart development.^78^ Currently, the application of *C. elegans* spatial transcriptomics is in its infancy with only three publications known to date,^74^^,^^79^^,^^80^ none of which have been conducted in the context of microgravity. Similarly, cell-specific proteomics methodologies that permit analysis of body wall muscles, neurons, and pharyngeal muscles have been developed^81^ but are yet to be exploited in the context of microgravity.

In the absence of high-throughput spatiotemporal techniques, spatiotemporal insight is traditionally and routinely gained in *C. elegans* through the utility of GFP.^38^^,^^82^ Capitalizing on the nematodes transparent body, GFP is “tagged” to a gene/protein of interest to gain information on expression patterns and subcellular localization *in vivo*. In the context of simulated microgravity, Zhao and colleagues demonstrated the temporal induction of oxidative stress and the underlying mechanisms.^82^ In the context of microgravity, the utility of GFP helped to demonstrate that treatment with RNAi works as effectively in space as it does on Earth in multiple tissues.^38^ Most recently, as part of the MME, GFP was exploited to monitor neuronal responses to microgravity.^47^

In summary, despite the potential promise of these emerging methods for transforming our mechanistic understanding of spaceflight (patho)physiology, these methods must be trialed for effectiveness in the spaceflight environment, much like RNAi technology was.^38^

## Conclusion

*C. elegans* represents a cheap and feasible organism to study the mechanisms regulating phenotypic adaptations in response to microgravity. We highlight that the application of omics analysis to spaceflight research has identified many ways that *C. elegans* respond to microgravity, at the level of the transcriptome, proteome, and epigenome. Notably, microgravity elicits transcriptional responses with microgravity robustly regulating genes related to muscle locomotion and metabolism (Figure 1). Similarly, proteomic changes in microgravity represent robust declines in cytoskeletal and metabolic proteins, which may underpin the pathophysiological phenotype associated with microgravity, particularly muscle decline. Importantly, these major transcriptional signatures are reproducible despite substantial differences in culturing conditions. Future work should consider using emerging omics approaches, such as multi-omics and spatial transcriptomics, to unravel tissue-specific spatiotemporal responses to microgravity in *C. elegans*. Where possible, such experiments should adopt, or at least consider, consortium-devised omics-related working pipelines in order to standardize experimental design, such as those developed by the International Standards for Space Omics Processing. In turn, this will facilitate future comparative omics investigations, thus extracting as much insight as possible from rare space omics experiments. This will lead to the identification of the fundamental mechanisms regulating (mal)adaptations to microgravity, benefiting health while in space and on Earth (e.g., aging, diabetes).
